# Association of CYP2C19 genetic polymorphisms with dyslipidemia and ischemic cerebrovascular disease in the mongolian population: A case-control study

**DOI:** 10.1097/MD.0000000000050807

**Published:** 2026-10-02

**Authors:** Gejile Hu, Jianhui Zhang, Rui Shi, Jingyang Yu, Shiyang Wang, Baoqiang Hu, Pengnan Bao, Yue Wang

**Affiliations:** a Department of Neurology, Tongliao People’s Hospital, Tongliao, China; b Department of Neurology, Baotou Central Hospital, Baotou, China.

**Keywords:** CYP2C19 gene polymorphism, dyslipidaemia, ischemic cerebrovascular disease, Mongolian population

## Abstract

This study investigates the role of cytochrome P450 2C19 (CYP2C19) gene polymorphisms and dyslipidaemic metabolism in ischemic cerebrovascular disease among Mongolian patients, analyzing their interaction with conventional risk factors. This case-control study enrolled 300 patients with ischemic cerebrovascular disease: 150 Mongolian patients (observation group) receiving CYP2C19 genotype-guided treatment and 150 Han Chinese patients (control group) undergoing standard care. Baseline characteristics, lipid profiles, CYP2C19 genotypes, NIHSS, and activities of daily living scores were analyzed. Baseline characteristics and CYP2C19 genotype distributions showed no significant differences between groups (*P* > .05). Multivariate logistic regression revealed that the poor metaboliser phenotype significantly increased the risk of ischemic cerebrovascular disease odds ratio (OR) = 2.31, 95% confidence interval: 1.25–4.26, *P* = .008), alongside hypertension (OR = 1.87, *P* = .043) and diabetes mellitus (OR = 2.12, *P* = .024). Dyslipidaemia prevalence was marginally but not significantly higher across genotypes in the Mongolian group. After 3 months of treatment, the genotype-guided observation group exhibited significantly greater reductions in total cholesterol and low-density lipoprotein cholesterol, and a slight increase in high-density lipoprotein cholesterol (*P* < .05). Furthermore, the observation group demonstrated significantly superior neurological recovery National Institutes of Health Stroke Scale and improved daily functioning (ADL) compared to the control group (*P* < .001). CYP2C19 poor-metaboliser status is associated with an increased risk of ischemic cerebrovascular disease in the Mongolian population and may contribute to dyslipidaemia and treatment response heterogeneity. Individualised intervention guided by CYP2C19 genotyping offers potential benefits in improving lipid profiles, neurological function, and short-term outcomes, serving as a valuable tool for precision medicine in this population.

## 1. Introduction:

Stroke is a neurological disorder caused by sudden localized disruption of cerebral blood flow, ranking among the primary causes of disability and mortality. Ischemic stroke (IS) accounts for 70% to 80% of all stroke types. The accelerating global aging population, coupled with inadequate control of risk factors in some stroke patients, has led to rising incidence and prevalence rates. Cerebral infarction carries a high recurrence rate, with the risk of a recurrent stroke within 3 months of the initial event estimated at approximately 5% to 10%.^[1–3]^ The 1-year recurrence rate stands at 17%, and the cumulative 5-year recurrence rate reaches 41%.^[4]^ Subsequent cerebral infarction episodes markedly increase disability rates and mortality among patients. Clopidogrel is currently a commonly used drug for secondary prevention of ischemic stroke (IS), though its efficacy and safety exhibit significant variability. As a prodrug, clopidogrel belongs to the P2Y12 inhibitor class and is metabolically activated by the highly polymorphic hepatic cytochrome CYP2C219 enzyme. Secondary prevention efficacy is compromised in carriers of cytochrome P450 2C19 (CYP2C19) loss-of-function alleles, affecting 25% of Caucasian patients and 60% of Asian patients.^[5]^ The high degree of polymorphism in the CYP2C19 gene is primarily attributable to the mutant alleles *1 and *2, *2, and *3, particularly the CYP2C19 *2 and *3 alleles. These are categorized as loss-of-function variants, leading to significantly reduced drug metabolism capacity in carriers. Insufficient production of clopidogrel’s active metabolite results in diminished antiplatelet effects, potentially even resistance, thereby increasing the risk of thrombotic events and recurrent stroke.^[6]^ Concurrently, studies indicate an association between CYP2C19 genotypes and lipid regulation. Functional loss variants may induce dyslipidaemia, thereby promoting atherosclerotic development and exacerbating ischemic cerebrovascular disease.^[7]^

Current research on the relationship between CYP2C19 gene polymorphisms and ischemic cerebrovascular disease, both domestically and internationally, has predominantly focused on Han Chinese or other major ethnic populations. Systematic data reporting on the Mongolian ethnic group remains relatively scarce. As one of China’s principal minority ethnic groups, the Mongolians possess unique genetic backgrounds, environmental influences, and dietary factors. Consequently, CYP2C19 gene polymorphisms and CYP2C19 enzyme activity exhibit certain differences compared to Han Chinese populations.^[8]^ Their high-fat, high-protein dietary habits may confer distinct characteristics regarding lipid metabolism and atherosclerosis risk compared to Han Chinese. Utilising CYP2C19 genetic testing to guide antiplatelet drug selection facilitates the development of personalized treatment regimens, thereby reducing the incidence and recurrence rates of IS. Therefore, this study employs retrospective analysis to conduct a systematic statistical examination of CYP2C19 gene polymorphisms, dyslipidaemia, and the occurrence and progression of ischemic cerebrovascular disease within the Mongolian population. It further explores the interaction between these factors and traditional risk factors (such as body mass index [BMI], blood pressure levels, and history of diabetes). It is hoped that this study will provide new theoretical support for the early identification and precise prevention and treatment of high-risk individuals among the Mongolian population, offer reference for formulating personalized intervention strategies for ischemic cerebrovascular disease among domestic ethnic minorities, and lay the groundwork for establishing a stroke prevention and control system from a gene-environment interaction perspective in the future.

## 2. Materials and methods

### 2.1. General information

This study selected 300 patients diagnosed in the Neurology Department of Tongliao People’s Hospital between January and December 2024 who met the inclusion and exclusion criteria. Subjects were categorized by ethnicity: 150 Mongolian patients were assigned to the observation group, comprising 92 males and 58 females. with a mean age of 62.4 ± 9.6 years and a BMI of 25.8 ± 3.2 kg/m^2^. The remaining 150 Han Chinese patients formed the control group, comprising 90 males and 60 females, with a mean age of 61.9 ± 10.1 years and a BMI of 25.5 ± 3.4 kg/m^2^. Comparison of general characteristics between the 2 groups revealed no statistically significant differences (*P* > .05) (see Table 1). This study was reviewed and approved by the Medical Ethics Committee of Tongliao People’s Hospital (Approval No. Tongyan Shen [2023] 25; approved on September 11, 2023).

**Table 1:** Comparison of general data.

Group	sexual distinction (male/female)	Age (yr)	BMI (kg/m^2^)	Hypertension n(%)	Diabetes n(%)	Hyperlipidemia n(%)
Observation	92/58	64.7 ± 11.2	24.9 ± 3.5	88 (58.7%)	42 (28.0%)	65 (43.3%)
Control	90/60	65.3 ± 10.6	25.2 ± 3.2	90 (60.0%)	45 (30.0%)	68 (45.3%)
*T*	–	0.42	0.76	0.08	0.18	0.21
*P*	–	.67	.45	.78	.67	.65

BMI = body mass index.

#### 2.1.1. Inclusion and exclusion criteria

##### 2.1.1.1. Inclusion criteria

All patients met the Diagnostic Criteria for Ischemic Cerebrovascular Disease, confirmed by imaging (CT or MRI) and clinical symptoms.

Patient age range was 40 to 82 years;

All patients underwent CYP2C19 genotyping via point-of-care testing prior to enrollment;

Patients with a history of first-episode or previous stroke who had been stable for over 6 months, with no evidence of acute hemorrhagic disease;

Voluntary participation in the study, with signed informed consent; ability to adhere to regular medication and follow-up appointments; willingness to cooperate with follow-up and monitoring procedures.

##### 2.1.1.2. Exclusion criteria

Patients with concomitant severe conditions: Exclude those with severe heart failure, hepatic or renal insufficiency, active gastrointestinal hemorrhage, malignant tumors, or other serious systemic diseases.

Exclude patients with a history of intracranial hemorrhage, gastrointestinal bleeding, coagulation disorders, or long-term anticoagulant therapy.

Drug contraindications: Patients with allergy or intolerance to aspirin, clopidogrel, ticagrelor, statins, calcium channel blockers, or metformin shall not be included.

Patients receiving other antiplatelet agents (e.g., clopidogrel, ticagrelor) within 1 month prior to enrollment.

Incomplete genetic testing: Exclude those who have not completed CYP2C19 genotyping, or whose test results are incomplete or inconclusive.

Special populations: Pregnant or breastfeeding women, or patients with mental disorders affecting cognitive judgment. Also, exclude those with poor compliance: Patients unable to cooperate with the study drug regimen and regular follow-up visits.

#### 2.1.2. CYP2C19 genotypes

In this study, genotyping was performed using the CYP2C19 Genetic Testing Kit, based on fluorescence PCR, and the corresponding Wanfo iReader 3000 point-of-care testing analyzer, both manufactured by Guangzhou Wondfo Biotech Co., Ltd. All testing procedures were conducted strictly in accordance with the manufacturer’s instructions. Briefly, 200 μL of peripheral venous whole blood was collected from each patient and added to the sample-processing solution. After nucleic acid extraction, PCR amplification, and fluorescence detection, the instrument automatically interpreted the genotyping results for the *1, *2 and *3 alleles. Quality control was performed for each testing batch using negative, positive and blank controls. The intra-assay coefficient of variation was < 3%, and the inter-assay coefficient of variation was < 5%, supporting the accuracy and reproducibility of the test results.

Each patient’s *1, *2, and *3 alleles were recorded, with all genotypes (including 1/*3, *2/*3, and *3/3) fully documented. Patients were categorized as rapid, intermediate, or poor metabolisers based on their genotype, where the rapid metaboliser genotype is *1/*1. Intermediate metaboliser (IM) genotypes comprised *1/*2 and *1/*3; poor metaboliser (PM) genotypes comprise *2/*2, *2/3, or 3/*3. Although the detection rates for 1/*3, *2/*3, and *3/*3 genotypes were low in this study, all genotypes (*1/*1, *1/*2, *1/*3, *2/*2, *2/*3, *3/*3) were fully documented. Subsequent analyses first applied Hardy-Weinberg equilibrium testing to all genotypes before grouping subjects into rapid, intermediate, and PM cohorts based on metabolic function for statistical analysis, ensuring data integrity and analytical rigor.

### 2.2. Treatment methods

Upon admission, standardized secondary prevention therapy is initiated, comprising aspirin 100 mg daily to reduce thrombogenesis, atorvastatin calcium 20 mg daily to optimize the lipid profile, amlodipine 5 to 10 mg daily to control blood pressure, and metformin 500 mg to optimize glucose metabolism. Following initial treatment, the regimen is tailored based on CYP2C19 genetic testing results. For rapid metabolizers, employ the standard protocol: dual antiplatelet therapy with clopidogrel 75 mg daily combined with aspirin 100 mg daily for 21 days, transitioning thereafter to monotherapy antiplatelet treatment. For patients identified as PM , administer ticagrelor 60 mg twice daily in combination with aspirin 100 mg daily for antiplatelet therapy.

### 2.3. Observation indicators

Data collection for enrolled patients included blood pressure, blood glucose, BMI, lipid profile triglycerides (TG), total cholesterol (TC), high-density lipoprotein cholesterol (HDL-C), low-density lipoprotein cholesterol (LDL-C), neurological deficit status National Institutes of Health Stroke Scale (NIHSS) score, and activities of daily living (ADL) score.

For all patients, 5 mL of fasting antecubital venous blood was collected between 08:00 and 12:00 on the morning after admission, following an 8–12 hours fast. Blood samples were collected into EDTA anticoagulant tubes and centrifuged at 3000 r/min for 10 minutes within 30 minutes of collection to separate plasma. Plasma samples were then stored uniformly at−80°C until analysis. Lipid levels were measured using a Roche Cobas 8000 automated biochemical analyzer with matched original reagents. The intra-assay and inter-assay coefficients of variation were < 2% and < 4%, respectively, and test results were recorded to 2 decimal places. Dyslipidemia was diagnosed according to the 2023 Chinese Guidelines for the Management of Dyslipidemia in Adults and was defined as the presence of at least one of the following abnormalities: fasting TG > 2.3 mmol/L, TC > 5.2 mmol/L, LDL-C > 3.4 mmol/L, or HDL-C < 1.0 mmol/L. Each patient’s baseline lipid profile was recorded, with subsequent measurements taken at 1, 3, and 6 months posttreatment to analyze dynamic lipid changes. Additionally, clinical data including medical history (hypertension, coronary heart disease), diabetes history, and smoking/alcohol consumption history were collected. Blood pressure, blood glucose, and lipid levels were monitored weekly throughout follow-up and treatment.

### 2.4. Statistical methods

Statistical analysis was performed using 26.0 software. For quantitative data conforming to normal distribution, results are presented as mean standard deviation ($\bar{X}\pm s$); f ± non-normally distributed data, median (interquartile range) is used. Intergroup comparisons employed independent samples t-tests or Mann-Whitney *U* tests. Categorical data were presented as counts and percentages, with intergroup comparisons conducted using chi-square (*χ^2^*) tests. The association between genotypes and dyslipidemia or ischemic cerebrovascular disease occurrence was analyzed using univariate and multivariate logistic regression, calculating odds ratios (OR) and 95% confidence intervals (CI). Continuous variables underwent univariate analysis of variance (ANOVA), with post hoc post hoconferroni corrections applied where necessary. To assess population representativeness of enrolled samples using the Hardy-Weinberg equilibrium test, all CYP2C19 genotypes were included. Actual and expected counts for each genotype were calculated alongside *χ^2^* values; *P* > .05 > cated H-W equilibrium. *P* < .05 was considered statistically significant. All data in tables were rounded to 2 decimal places, with *t* values and *P* values presented in the results tables. Multivariate logistic regression models were employed to analyze the effects of age, gender, BMI, and diabetes history on the occurrence of dyslipidemia and ischemic cerebrovascular disease.

## 3. Results

### 3.1. General information comparison

There were no statistically significant differences between the observation group (Mongolian ethnicity) and the control group (Han ethnicity) in terms of gender, age, BMI, or preexisting conditions such as hypertension, diabetes mellitus, or hyperlipidemia (*P* > .05). This indicates that the intragroup comparisons within each cohort were statistically significant (see Table 1).

### 3.2. CYP2C19 genotype distribution and its association with dyslipidemia

Hardy-Weinberg equilibrium testing for CYP2C19*1, CYP2C19*2 and CYP2C19*3 genotypes revealed genetic equilibrium within the population, with data derived from the same Mendelian population (*P* > .05). Grouping was based on metabolic type. The observation group’s CYP2C19 genotype distribution: *1/*1 (56 cases), *1/*2 (62 cases), *1/*3 (2 cases), *2/*2 (27 cases), *2/*3 (1 case), *3/3 (0 cases); The control group’s genotype distribution was: *1/*1 in 60 cases, *1/*2 in 60 cases, *1/*3 in 2 cases, *2/*2 in 26 cases, *2/*3 in 2 cases, and *3/*3 in 0 cases. Hardy-Weinberg equilibrium testing was conducted for all CYP2C19 genotypes across both groups. Results indicated equilibrium in both cohorts (*P *> .05), confirming the sample’s robust population representativeness (see Table 2).

**Table 2:** Hardy-Weinberg equilibrium test for CYP2C19 genotypes in the observation and control groups.

Group	Genotype	Actual observations	Expected number E	*χ^2^*	Allele frequency (*1/ *2/ *3)
Observation (n = 150)	*1/*1	56	51.6	0.375	0.587/ 0.390/ 0.023
	*1/*2	62	67.8	0.496	
	*1/*3	2	2.7	0.189	
	*2/*2	27	22.8	0.774	
	*2/*3	1	2.4	0.729	
	*3/*3	0	0.015	0.015	
Free degree/ *P* value	–	–	–	5/ .80	–
Control (n = 150)	*1/*1	60	55.0	0.455	0.606/ 0.377/ 0.017
	*1/*2	60	69.9	1.322	
	*1/*3	2	2.8	0.229	
	*2/*2	26	20.4	1.573	
	*2/*3	2	1.8	0.022	
	*3/*3	0	0.015	0.015	
free degree/ *P*	–	–	–	5/ .87	–

### 3.3. CYP2C19 genotype and incidence of ischemic cerebrovascular disease

The incidence rates of IS/ transient ischemic attack (TIA) across genotypes in the observation group were as follows: *1/*1: 26.8% (15/56), *1/*2 38.7% (24/62), *1/*3 50.0% (1/2), *2/*2 59.3% (16/27), *2/*3 100.0% (1/1), *3/*3 (0/0); In the control group, the incidence rates of IS/TIA for each genotype were: *1/*1 26.7% (16/60), *1/*2 38.3% (23/60), *1/*3 50.0% (1/2), *2/*2 57.7% (15/26), *2/*3 50.0% (1/2), *3/*3 (0/0). After grouping by metabolic phenotype, the incidence rates of IS/TIA in the observation group were 26.8% (15/56) for fast metabolizers, 39.0% (25/64) for IM and 60.7% (17/28) for slow metabolizers. In the control group, these were 26.7% (16/60), 38.7% (24/62), and 57.1% (16/28) respectively (see Table 3). Differences between genotype groups were not statistically significant (*P *> .05).Multivariate logistic regression analysis indicated that patients with the low-metabolism genotype (including *2/*2 and *2/*3) may exhibit an increased risk of ischemic cerebrovascular disease (OR = 2.31, 95% CI 1.25 to 4.26, *P* = .008), suggesting this genotype (combination) may represent a potential risk factor. However, no significant differences were observed in overall genotype distribution comparisons (see Table 4).

**Table 3:** Comparison of IS/TIA incidence across different genotypes and metabolic phenotypes.

Genotype/Metabolic phenotype	Observation group IS/TIA (n = 148)	Control group (n = 150)	*P*
Genotype			
*1/*1	15/56 (26.8%)	16/60 (26.7%)	>.05
*1/*2	24/62 (38.7%)	23/60 (38.3%)	>.05
*1/*3	1/2 (50.0%)	1/2 (50.0%)	>.05
*2/*2	16/27 (59.3%)	15/26 (57.7%)	>.05
*2/*3	1/1 (100.0%)	1/2 (50.0%)	>.05
*3/*3	0/0 (−)	0/0 (–)	-
Metabolic phenotype			
RM	15/56 (26.8%)	16/60 (26.7%)	>.05
IM	25/64 (39.0%)	24/62 (38.7%)	>.05
PM	17/28 (60.7%)	16/28 (57.1%)	>.05

IS = ischemic stroke, PM = poor metabolizer, RM = rapid metabolizer, TIA = transient ischemic attack.

**Table 4:** CYP2C19 genotype and incidence of ischemic cerebrovascular disease.

Genotype	Observation group IS/TIA n(%)	Control group IS/TIA n(%)	*t*	*P*
RM	15/56 (26.8%)	16/60 (26.7%)	0.00	.99
IM	25/64 (39.1%)	24/62 (38.7%)	0.01	.97
PM	18/30 (60.0%)	16/28 (57.1%)	0.08	.81
Multi-factor logistic OR (95%CI)	2.31(1.25–4.26)	–	–	.01

RM = rapid metabolizer genotype, IM = Intermediate metabolizer genotypes comprised, PM = Poor metaboliser genotypes.

Subsequently, multivariate logistic regression analysis was conducted on variables demonstrating statistical significance in the univariate analysis (age ≥ 60 years, hypertension, diabetes mellitus, hypometabolic phenotype) (see Table 5). Results indicated that the hypometabolic phenotype significantly increased the risk of ischemic cerebrovascular disease (OR = 2.31–95% CI: 1.25–4.26, *P* = .008). Hypertension (OR = 1.87, 95% CI: 1.02–3.41, *P* = .043) and diabetes mellitus (OR = 2.12, 95% CI: 1.11–4.05, *P* = .024) were also identified as independent risk factors. Age ≥ 60 years showed no significant difference in multivariate analysis (as shown in Table 6) (*P > *.05). This suggests that CYP2C19 PM may represent a potential risk factor for ischemic cerebrovascular disease, acting in conjunction with hypertension and diabetes. Clinicians should therefore consider this genotype when conducting individualized risk assessments within the Mongolian population.

**Table 5:** Univariate analysis: factors associated with ischemic cerebrovascular disease.

Variable	Patient (n)	Disease-free (n)	χ^2^	*P*	Whether to include
Age ≥ 60 yr old	45	20	7.56	.006	1
Hypertension	52	28	5.32	.021	1
Diabetes	33	10	6.45	.011	1
Hyperlipidemia	24	18	2.11	.146	0
Smoke	28	20	1.32	.251	0
Drink	21	16	0.87	.351	0
Low metabolic type	18	16	4.12	.042	1

Include it in the multifactor analysis; 2:Not included.

**Table 6:** Results of multivariate logistic regression analysis.

Variable	OR value	95%CI	*P*
PM	2.31	1.25–4.26	.008
Hyperlipidemia	1.87	1.02–3.41	.043
Diabetes	2.12	1.11–4.05	.024
Age ≥ 60 yr old	1.42	0.78–2.59	.256

PM = poor metaboliser genotypes.

### 3.4. Comparison of lipid-related indicators

The incidence of dyslipidemia was marginally higher across all genotypes in the observation group compared to the control group, though differences were not statistically significant (*P *> .05). This suggests a slight, nonsignificant increase in dyslipidemia prevalence among Mongolian individuals with CYP2C19 low-metabolism genotypes, indicating a mild positive trend (as shown in Table 7). Following 3 months of treatment, the observation group exhibited greater reductions in TC and LDL-C, alongside a slight increase in HDL-C (as shown in Table 8). These differences were statistically significant (*P *< .05).

**Table 7:** CYP2C19 genotypes and incidence of dyslipidaemia.

Genotype	Observation n(%)	Control n(%)	*t*	*P*
RM	22/56 (39.3%)	23/60 (38.3%)	0.01	.92
IM	35/64 (55.0%)	33/62 (53.2%)	0.03	.86
PM	20/28 (73.3%)	20/28 (71.4%)	0.04	.84

Dyslipidemia is defined as the presence of any one of the following abnormal lipid markers: TG > 2.3 mmol/L, TC > 5.2 mmol/L, LDL-C > 3.4 mmol/L, or high-density HDL-C < 1.0 mmol/L.

HDL-C = High-Density Lipoprotein Cholesterol, LDL-C = Low-Density Lipoprotein Cholesterol, TC = Total Cholesterol, TG = Triglyceride.

**Table 8:** Comparison of lipid-related indicators.

Group	TG (mmol/L)	TC (mmol/L)	LDL-C (mmol/L)	HDL-C (mmol/L)
Enrollment	1.98 ± 0.72	5.52 ± 0.85	3.45 ± 0.64	1.15 ± 0.22
Observation (The third mouth)	1.55 ± 0.61	4.42 ± 0.78	2.67 ± 0.53	1.28 ± 0.21
Control (The third mouth)	1.65 ± 0.65	4.78 ± 0.82	2.91 ± 0.57	1.22 ± 0.23
*t*	2.03	3.41	3.12	2.02
*P*	.045	.001	.002	.046

HDL-C = High-Density Lipoprotein Cholesterol, LDL-C = Low-Density Lipoprotein Cholesterol,TC = Total Cholesterol,TG = Triglyceride.

### 3.5. Comparison of NIHSS and ADL scores before and after treatment

Changes in NIHSS and ADL scores in the observation and control groups at enrollment and at the 3-month follow-up are shown in Table 9. The NIHSS score was used to assess the severity of neurological deficits, with higher scores indicating more severe impairment. The ADL score was used to evaluate ADL , with higher scores indicating greater independence. At the 3-month follow-up, the observation group showed greater improvement in neurological function and ADL than the control group, and the between-group differences were statistically significant (*P* < .001).

**Table 9:** Comparison of NIHSS and ADL scores before and after treatment.

Group	NIHSS (enrollment)	NIHSS (The third mouth)	ADL (Enrollment)	ADL (The third mouth)
Observation	7.8 ± 3.2	4.2 ± 2.1	56.2 ± 12.5	76.8 ± 10.4
Control	7.5 ± 3.0	4.8 ± 2.3	57.0 ± 11.8	72.5 ± 11.2
*t*	8.76		7.92	
*P*		.000		.000

ADL = activities of daily living, NIHSS = national institutes of health stroke scale.

## 4. Discussion

Cytochrome P450 enzymes (CYP450) constitute a subfamily of heme oxygenase enzymes, with the CYP1, CYP2, and CYP3 subfamilies playing a decisive role in human drug metabolism.^[9]^ As a key member of the cytochrome P450 enzyme family, CYP2C19 plays a vital role in metabolizing numerous drugs and both endogenous and exogenous substances, exhibiting significant metabolic activity in the hepatic metabolism of exogenous drugs.^[10]^ Certain drugs require metabolism by hepatic cytochrome P450 enzymes to generate active metabolites before exerting their effects. Clopidogrel exemplifies such a prodrug: approximately 85% of clopidogrel in the bloodstream is inactivated by esterases in its parent form, with only 15% of the prodrug being converted into the active metabolite. This metabolite irreversibly binds to the platelet P2Y12 receptor, inhibiting it and ultimately exerting an antiplatelet effect.^[11]^ The CYP2C19 gene exhibits polymorphism. During its regulation of CYP2C19 enzyme activity in the human body, it is influenced by its own loss-of-function mutations, leading to altered enzyme activity. Consequently, different biological individuals or ethnic groups exhibit varying genetic effects on drug metabolism. These differing metabolic phenotypes result in variable drug concentrations among individuals. This process readily gives rise to adverse drug reactions, significantly compromising medication safety.^[12]^ Therefore, prior to administering antiplatelet agents such as clopidogrel for IS, consideration must be given to the impact of genetic polymorphisms on drug metabolism. By testing for these polymorphisms, one can ascertain an individual’s genetic metabolic phenotype. Appropriate drug selection and dose adjustment based on this phenotype can enhance clinical treatment efficacy, ensuring both safe and effective medication while achieving more personalized, precision-based therapy for the patient.^[13]^

To the present day, at least 35 CYP2C19 alleles have been identified. Among these, the CYP2C19*2 and CYP2C19*3 alleles result in marked resistance to clopidogrel, poor antiplatelet aggregation efficacy, and an increased probability of recurrent ischemic events, severely compromising treatment outcomes.^[14,15]^ Based on their capacity for metabolizing drugs, CYP2C19 enzymes commonly exhibit 3 phenotypes: extensive metabolizers (EM), IM, and PM. Homozygous mutations result in a complete loss of metabolic activity, while heterozygous mutations cause reduced enzyme activity, leading to a progressive decrease in clopidogrel metabolism capacity across these phenotypes. The extensive metabolizer (EM) genotype corresponds to the wild-type CYP2C19*1 homozygote; IM are heterozygous for the mutation, with genotypes including CYP2C19*1/*2 and CYP2C19*1/*3; PM are homozygous for the mutation, encompassing CYP2C19*2/*2, CYP2C19*3/*3, and CYP2C19*2/*3. Given China’s vast territory, significant variations in environmental factors and dietary habits exist. Differences in CYP2C19 gene polymorphisms and CYP2C19 enzyme activity are also observed among different ethnic groups. Investigating and understanding these variations may prove beneficial in guiding individualized clinical medication strategies. According to relevant studies, the probabilities of carrying a loss-of-function allele among Europeans, Africans, and Asians are 28%, 42%, and 59%, respectively. Furthermore, other research indicates that Asian populations are more prone to acquiring GF*2 and GF*3 allele mutations, resulting in reduced drug metabolism rates.^[16]^ These factors collectively contribute to increased adverse drug reactions, including recurrent vascular events and hemorrhage, during clopidogrel therapy for IS. This study analyzed the association between CYP2C19 gene polymorphisms and dyslipidemia, as well as ischemic cerebrovascular disease (IS, TIA) in the Mongolian population. Findings revealed that patients with the low-metabolism genotype exhibited a heightened risk of ischemic cerebrovascular disease (OR = 2.31, 95% CI: 1.25–4.26, *P* = .008), suggesting this genotype represents a potential risk factor significantly elevating the risk of ischemic cerebrovascular disease. This genotype’s influence should be considered during individualized risk assessment in the Mongolian population. Research indicates that variability in fasting blood glucose (FBG) correlates with future cardiovascular events. Furthermore, each 20 mm Hg increase in systolic blood pressure and each 10 mm Hg increase in diastolic blood pressure may double the risk of death from stroke, heart disease, or other vascular conditions. These findings underscore the critical importance of screening for and treating hypertension and diabetes in preventing such diseases.^[17]^ This study also identified hypertension (OR = 1.87, 95% CI: 1.02–3.41, *P* = .043) and diabetes (OR = 2.12, 95% CI: 1.11–4.05, *P* = .024) as independent risk factors for IS. CYP2C19 poor metabolism primarily affects antiplatelet drug metabolism, potentially diminishing clopidogrel efficacy. Conversely, ticagrelor reversibly blocks platelet P2Y12 receptors without requiring metabolic activation, rendering it particularly suitable for patients harboring CYP2C19 loss-of-function alleles.

Low-density lipoprotein cholesterol and TG have emerged as novel secondary prevention targets for stroke, while low-metabolism variants may further amplify dyslipidemia risks. Several randomized clinical trials, such as JELIS (Japanese EPA Lipid Intervention Study),^[18–20]^ RECATE-IT (Reducing Cardiovascular Events with Eicosanoid Isomerase Ethyl Ester Trial), STANCE (Study to Assess the Long-Term Outcomes of Epanova in Patients with Hypertriglyceridemia and High Cardiovascular Risk), and PROMPANCED (Preventing Cardiovascular Outcomes by Lowering TG in Diabetic Patients) trials, have demonstrated the potential benefits of TG reduction in decreasing recurrent major cardiovascular events among cardiovascular disease patients. Further studies indicate that the efficacy of dual antiplatelet therapy remains unaffected by low-density lipoprotein levels, with ticagrelor-aspirin demonstrating superiority over clopidogrel-aspirin in elevating non-low-density lipoprotein levels while treating stroke.^[21,22]^

This study found that following a 3-month period of personalized intervention, lipid-related parameters in the observation group demonstrated significantly greater improvement compared to the control group (*P* < .05). This suggests that CYP2C19 genotype-based personalized drug intervention can effectively optimize lipid regulation, providing evidence for the precise prevention and treatment of ischemic cerebrovascular disease. Intergroup comparisons of lipid-related indicators revealed that patients in the experimental group exhibited a greater increase in low-metabolism type LDL-C and a more pronounced decrease in HDL-C, with significant differences observed. The control group demonstrated a relatively lower incidence of dyslipidemia under the same genotype. Although CYP2C19 genotype distributions differed between the Mongolian and Han ethnic groups, no significant differences were noted. Therefore, the cause of dyslipidemia in the experimental group is currently considered to be exacerbated by the Mongolian population’s characteristic high-protein, high-fat dietary structure and lifestyle. Furthermore, during the observation period, neither group experienced drug-related adverse reactions such as hemorrhage, abnormal liver function, or elevated creatine kinase levels, indicating the adopted intervention protocol possesses favorable safety. In terms of clinical outcomes, changes in NIHSS and ADL scores before and after treatment showed that the observation group, which received genotype-guided individualized antiplatelet therapy and lipid-lowering intervention, achieved more pronounced improvement in neurological function. At the 3-month follow-up, the reduction in NIHSS scores was greater and the increase in ADL scores was more significant in the observation group than in the control group, with statistically significant between-group differences. These findings suggest that this comprehensive intervention regimen may help promote neurological recovery and improve ADL. Furthermore, they indicate that an individualized antiplatelet and comprehensive intervention strategy based on CYP2C19 genotype may have positive clinical significance for improving the prognosis of Mongolian patients with ischemic cerebrovascular disease.

The present study has several limitations. First, it was a retrospective, single-center study with a relatively small sample size and a limited follow-up duration. In addition, the experimental group mainly comprised Mongolian patients receiving CYP2C19 genotype-guided individualized therapy, whereas the control group consisted of Han patients receiving conventional clinical care, which may have introduced potential population and treatment-related bias. Nevertheless, this study suggests that the CYP2C19 genotype may contribute to differences in susceptibility to ischemic cerebrovascular disease and variation in treatment response among the Mongolian population, providing a theoretical basis for early screening and tailored intervention in high-risk Mongolian individuals. Future multicenter, large-scale, prospective studies involving multiethnic cohorts are needed to further clarify the clinical significance of the CYP2C19 genotype in dyslipidemia, cerebrovascular disease risk stratification, and the improvement of long-term prognosis.

## 5. Conclusion

Poor-metabolizer status for CYP2C19 may be associated with an increased risk of ischemic cerebrovascular disease in the Mongolian population and may also contribute to dyslipidemia and heterogeneity in treatment response. In this study, an individualized intervention strategy guided by CYP2C19 genotyping showed potential benefits in improving prognostic indicators, including lipid profiles, neurological function, and ADL. These findings suggest that CYP2C19 genetic testing may serve as a valuable reference for the early identification of high-risk individuals and for precision intervention in cerebrovascular disease among Mongolian populations. Nevertheless, multicenter, large-scale prospective studies involving diverse genetic backgrounds are warranted to further validate the clinical value of CYP2C19 genotyping in risk assessment, individualized treatment selection, and the improvement of long-term outcomes in cerebrovascular disease.

## Author contributions

**Data curation:** Gejile Hu, Jianhui Zhang.

**Project administration:** Gejile Hu, Pengnan Bao, Yue Wang.

**Supervision:** Gejile Hu, Rui Shi, Shiyang Wang, Baoqiang Hu, Yue Wang.

**Formal analysis:** Jianhui Zhang, Baoqiang Hu.

**Investigation:** Jianhui Zhang, Rui Shi, Jingyang Yu.

**Resources:** Jingyang Yu.

**Visualization:** Jingyang Yu, Pengnan Bao, Yue Wang.

**Validation:** Shiyang Wang.

**Methodology:** Baoqiang Hu.

**Software:** Baoqiang Hu.

**Conceptualization:** Yue Wang.

**Writing – original draft:** Yue Wang.

**Writing – review & editing:** Gejile Hu, Rui Shi, Shiyang Wang, Pengnan Bao, Yue Wang.
